# Investigation of the hydration process and biological activity of a novel nanosilver incorporated dicalcium silicate based retrograde filling material

**DOI:** 10.7717/peerj.14632

**Published:** 2023-02-01

**Authors:** Teena Dsouza, Aditya Shetty, Sudarshan Kini, Veena Shetty, Shama Rao, Rajesha Payaradka, Nijil Satheesan, Neevan Dsouza, Heeresh Shetty

**Affiliations:** 1 AB Shetty Memorial Institute of Dental Sciences, Nitte (Deemed to be University), Mangalore, India; 2 Nitte University Centre for Science and Educational Research, Nitte (Deemed to be University), Mangalore, India; 3 KS Hegde Medical Academy, Nitte (Deemed to be University), Mangalore, India; 4 Texas A&M University, Texas, United States of America; 5 Nair Hospital Dental College, Mumbai, India

**Keywords:** Calcium silicate, Nanosilver, Biocompatibility, Retrograde filling

## Abstract

**Background:**

Although several materials have been used for retrograde filling following apical surgeries, there is no consensus on a single best material. Tricalcium silicate-based types of cement have been developed as root-end filling materials mainly due to tricalcium silicate’s hydraulic properties. However, its unfavorable setting characteristics and minimal antimicrobial properties have necessitated the introduction of new additives into the existing commercially available materials. To design an affordable product based on a dicalcium silicate with a shorter set time, minimal cytotoxic complications, and enhanced antibacterial activity, we developed a new endodontic cement from pure raw materials, intending to satisfy the prerequisites of ideal retrograde material.

**Methods:**

The composition of the experimental calcium silicate-based cement included the addition of calcium chloride and silver nanoparticles in varying concentrations. Structural characterization was carried out using energy dispersive analysis by X-rays using scanning electron microscope (EDAX SEM) and hydration characteristics were performed using an X-ray diffractometer (XRD). The experimental material was further evaluated for biocompatibility using MTT ([3-(4,5-dimethylthiazol-2-yl)-2,5-diphenyl-2H-tetrazolium bromide)assay and antibacterial activity was evaluated using an agar diffusion test against *Enterococcus faecalis*.

**Results:**

The structural characterization and hydration characteristics revealed that the experimental cement was dicalcium silicate based with favorable biocompatibility and enhanced antibacterial activity. Tricalcium silicate based mineral trioxide aggregate (MTA) also had favourable biocompatibility, however, its antibacterial activity was significantly decreased when compared to the novel cement.

**Conclusion:**

All hydraulic cements that are available in the dental market are predominantly tricalcium silicate-based materials. There has been no evidence in the literature to date wherein it has been explored whether a dicalcium silicate-based hydraulic cement can solely be used in root-end cavities. The findings of the study revealed a dicalcium silicate based retrograde filling material with favourable biocompatibility exhibited immediately as well as in the set samples. Incorporation of silver nanoparticles boosted the antibacterial activity when compared to that of ProRoot MTA. This material could potentially reinstate the usual hype created with tricalcium silicate types of cement since dicalcium silicate cements also exhibit similar properties.

## Introduction

Over the past two decades, calcium silicate hydraulic cements such as mineral trioxide aggregate (MTA) have been embraced for use in periapical surgeries (Wei et al., 2012). The excellent biocompatibility, bioactivity, and good sealing ability have made these attractive for use by endodontic researchers around the world to modify and better the existing materials (Hwang et al., 2009). Following the initial commercialization of MTA, a wide range of freshly formulated calcium silicate cements became accessible for clinical usage in endodontics. The primary components of these novel cements are tricalcium silicate and dicalcium silicate. Calcium silicate compounds gain instant strength when hydrated due to their hydraulic properties, and studies have demonstrated that these aggregates enhance the angiogenic and transcription elements, as well as pulpal fibroblasts and progenitors. Other properties include bioinductive abilities, which influence cell proliferation and differentiation, and the generation of hard tissue (Wei et al., 2012). MTA has been the ultimate touchstone material for use in apical surgeries. However, its unfavorable setting time and high cost make it an unappealing choice for endodontic applications (Dsouza, Bhandary & Srinivasan, 2016). The fabrication of a flawless, prototype repair material is still a long way off (Chaudhari et al., 2022). The ability of these biomaterials to exert antibacterial activity while maintaining their biological characteristics presents an additional difficulty (Janini et al., 2021). There is a paucity in the literature regarding such a prototype that fulfills most of the ideal criteria. With an aim to design an affordable product based on calcium silicate with a shorter set time, minimal cytotoxic complications, and enhanced antibacterial activity, we developed a new endodontic cement from pure raw materials, intending to satisfy the prerequisites of ideal retrograde material. The experimental cements made from calcium silicate also contained calcium chloride and silver nanoparticles in varying amounts. The use of calcium chloride improved the setting procedure (Dsouza et al., 2020a). Silver nanoparticles were added to the powder mixture to boost its antibacterial action (Dsouza et al., 2020b). Dental cements consisting of calcium silicate have an intricate setting process that is significantly influenced by their composition (Lim et al., 2019). To ascertain the phase composition in the current work, we first explored the hydration mechanism of this cement. Further, the biocompatibility and antibacterial activity were investigated and compared with those of a commercially available calcium silicate-based cement, ProRoot MTA (Dentsply, Charlotte, NC, USA).

## Materials & Methods

Materials used in this study are given in Table 1:

The solid-state reaction approach was used to combine the powder ingredients. The powder oxides were weighed with an analytical balance and manually mixed with 98 percent ethanol for at least two hours in a porcelain mortar. The final step was sintering the created powder in a hot air oven for 24 h at 100 °C. To get the requisite homogeneous powdered combination, the resultant powder was filtered through a 250-micron mesh.

### Preliminary tests for optimization of the product

Preliminary work was done to optimize the experimental powder by subjecting it to different temperatures of either 100 °C, 250 °C, 400 °C, or 600 °C. A further temperature increase was not attempted as silver has a melting point of 961.8 °C. Setting time was determined for each powder mixture, this indicated that the setting time was favorable at temperatures of 100 °C and 600 °C. A pilot study on antibacterial activity against *Enterococcus faecalis* at these temperatures showed a zone of inhibition of 3–4 mm only for the powder component which was heated to 100 °C. Therefore, this was taken as the experimental powder for further evaluation of these properties.

### Structural characterization and hydration characteristics

#### Energy dispersive analysis by X-rays (EDAX) scanning electron microscope (SEM) to observe the morphological chemical constitution

EDAX under the SEM (Zeiss Gemini; Zeiss, Jena, Germany) was carried out to observe the surface morphology of the final powder.

#### X-ray diffractometer (XRD) for hydration characteristics

The phase compositions of the unhydrated and hydrated compositions were analyzed using a high-resolution XRD (Empyrean Panalytical series-3 XRD; Malvern Panalytical Inc., Westborough, MA, USA) supplied with a Cu-K_a_ in the 2 *θ* range of 10°–45°. QualX software was used for the qualitative phase analysis of X-ray diffraction spectra for both the unhydrated and hydrated samples.

##### Groups.

Group 1: nanosilverdicalcium silicate cement (nano CS).

Group 2: MTA (White ProRoot MTA; Dentsply).

##### Determination of sample size.

Based on the pilot study, the standard deviation of final setting time in Group 1 = 1.823 min, standard deviation in Group 2 = 1.431 min, mean difference was 1.6, the effect size was 0.983, the alpha error was 5%, power was 80% for a two-sided test, and the samples per group was 16, *i.e.* a total of 32. This was calculated using nMaster software version 2.

**Table 1:** Powder oxides used in the study.

Powder components	Percentage (by weight)
Calcium oxide (Merck Life Sciences Pvt. Ltd., Bengaluru, Karnataka, India	60
Silicon dioxide (Loba Chemie Pvt., Ltd., Mumbai, Maharashtra, India	20
Aluminium dioxide (Merck Life Sciences Pvt. Ltd., Bengaluru, Karnataka, India	9.5
Bismuth oxide (Loba Chemie Pvt., Ltd., Mumbai, Maharashtra, India	10
Silver nanoparticles (SRL Chemical, Mumbai, Maharashtra, India	0.5
Liquid component	10
Calcium chloride (Loba Chemie Pvt., Ltd., Mumbai, Maharashtra, India	

### Evaluation of biocompatibility

Human gingival fibroblasts were used, which were established in Nitte University Center for Stem Cell Research and Regenerative Medicine. The cells were cultured in Dulbeco Modified Eagle Medium (DMEM), supplemented with 5% foetal bovine serum, 100 UmL^−1^ penicillin, 100L mL^−1^ streptomycin, and two mmol L^−1^ L-glutamine, at 37 °C in a humidified environment of 95% air and 5% CO_2_. Cement pellets were prepared by mixing the powder with the liquid. The cytotoxicity was tested by dividing it into two subgroups:

Subgroup 1: freshly mixed cement pellets immediately placed into cell cultures (*n* = 16).

Subgroup 2: set cement pellets after 24 h (*n* = 16).

Set pellets were prepared by placing the mixed pellets in a cell culture incubator for 24 h. The set pellet was checked with a dental explorer. The fibroblast cells were seeded into 96 well plates (2.7 × 10^4^ cells) and incubated for 24 h to allow adhesion. Then, 16 pellets were placed into the culture wells. The pellets were tested after 24 h, 48 h, and 72 h of inoculation for cell viability by MTT [3-(4,5-dimethylthiazol-2-yl)-2,5-diphenyl-2H-tetrazolium bromide] assay. A microplate reader for an ELISA was used to measure the spectroscopic absorbance at 630 nm.

### Antibacterial activity

Antibacterial activity was assessed by an agar diffusion test against *E. faecalis* (ATCC 29212). Following serial dilution, the turbidity of the bacterial suspension in brain heart infusion (BHI) broth was adjusted to 0.5 McFarland standard, approaching 1.0 × 10^7^ colony forming units (CFU)/mL. Cement specimens (10 mm × 3.0 mm, *n* = 16) were sterilized with ethylene oxide gas for 24 h before the experiment. A cotton swab was used to smear each bacterial suspension equally onto the medium’s surface in two planes. The cement specimens were mounted on an agar plate and incubated overnight at 37 °C in an aerobic condition after the inoculum dried. At three distinct locations, the diameters of the inhibitory halo generated around the disc were visually gauged using a sliding caliper with 0.1 mm precision. By deducting the specimen’s diameter (10 mm) from the sum of the halo’s three average measurements, the sizes of the inhibitory halo were determined.

## Results

### EDAX SEM analysis

**Figure 1:**
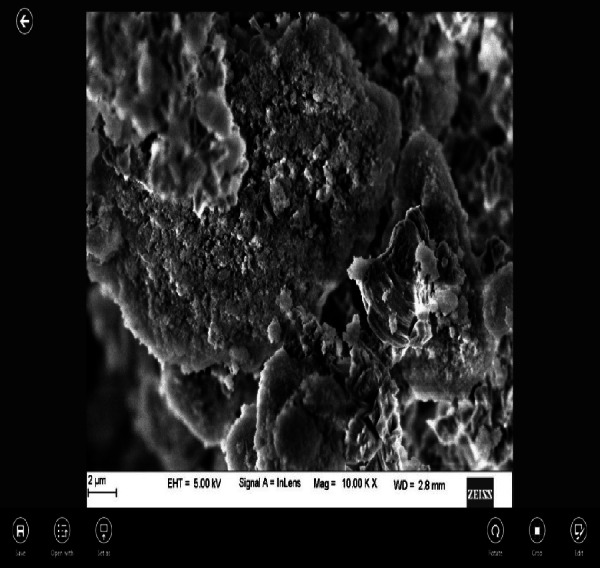
Surface morphology of the experimental cement at 2 µm magnification under SEM.

EDAX under scanning electron microscopic (SEM) analysis of nano CS showed that the particles were coarse and irregular (Fig. 1).

**Figure 2:**
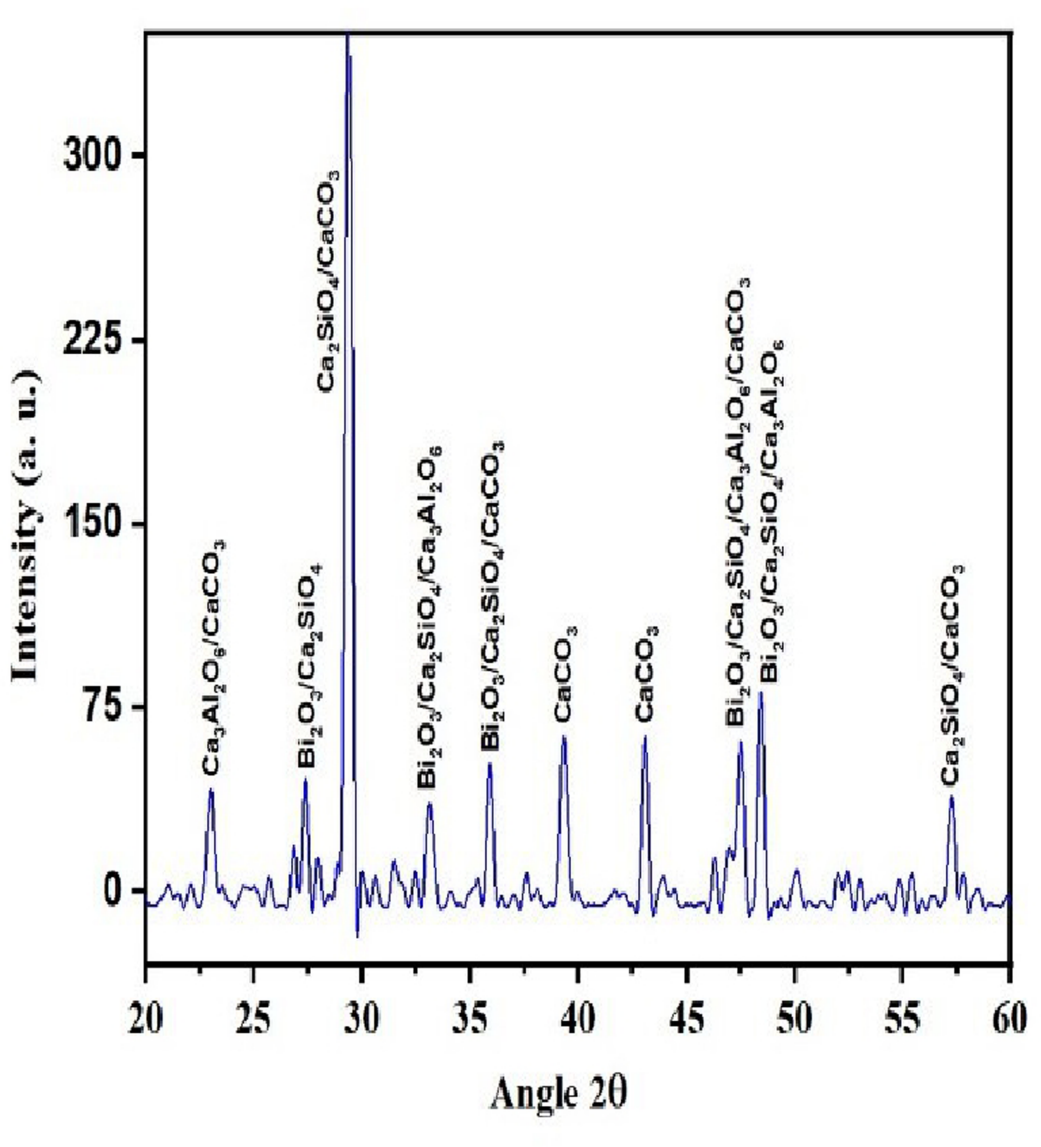
XRD spectra of the unhydrated powder sample.

**Figure 3:**
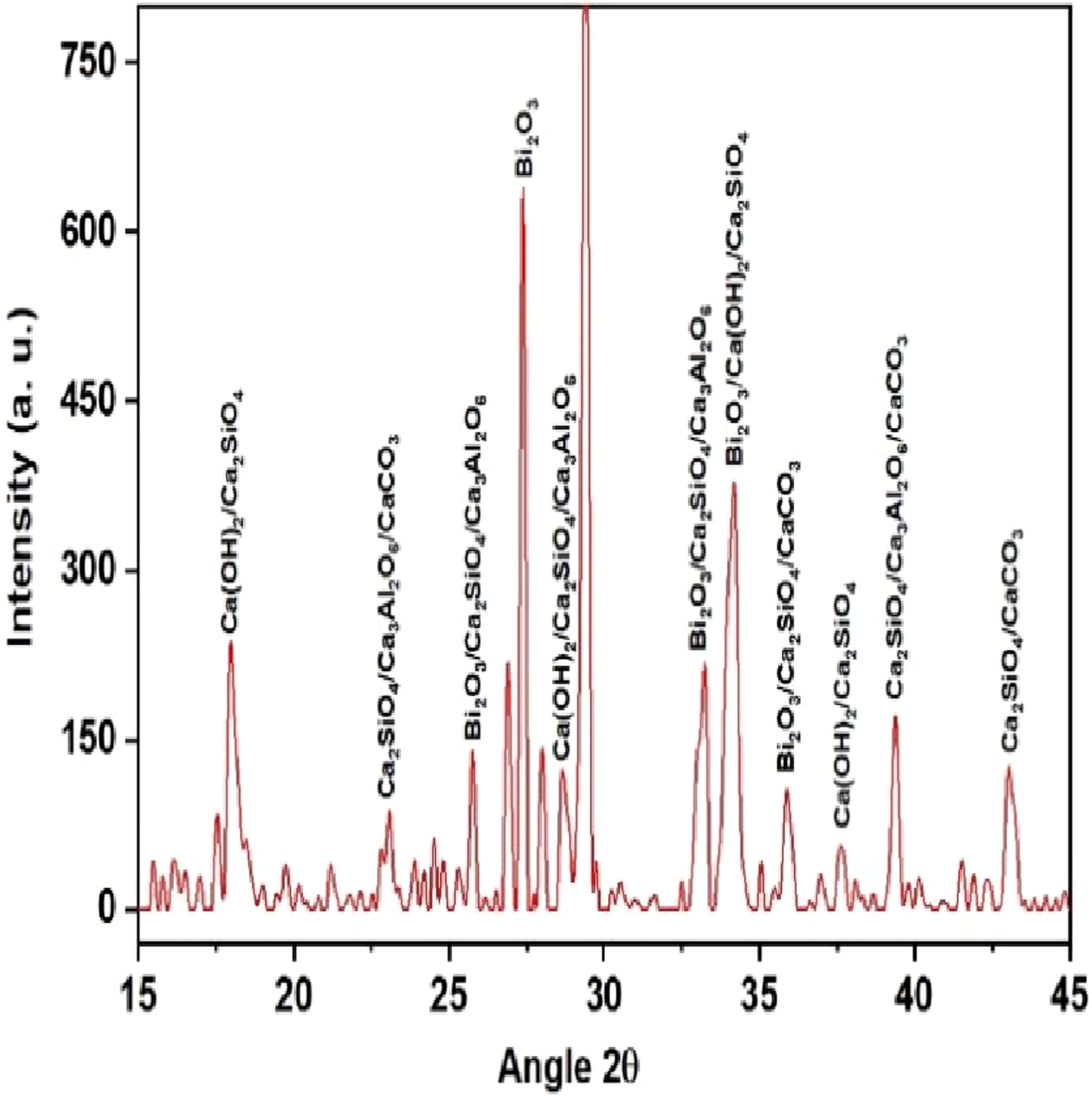
XRD spectra of the hydrated sample.

XRD analysis:

QualX software was used for the qualitative phase analysis of X-ray diffraction XRD spectra obtained for both the unhydrated powder and hydrated samples. The crystalline phases of the samples were identified using the structure information contained in the Crystallography Open Database (COD) (Gražulis et al., 2012). According to the COD the obtained phases in the unhydrated powder sample (Fig. 2) were bismite (Bi2O3) [COD: 00-101-004], dicalcium silicate (Ca2SiO4) [COD: 00-210-3316], tricalcium aluminate (Al2Ca3O6) [COD: 00-901-4359], and calcium carbonate (CaCO3) [COD: 00-101-0962]. The crystalline phases obtained in the case of the hydrated sample (Fig. 3) was as follows: bismite (Bi2O3) [COD: 00-901-2546], calcium hydroxide (Ca(OH)2) [COD: 00-100-1787], dicalcium silicate (Ca2SiO4) [COD: 00-210-3316], tricalcium aluminate (Al2Ca3O6) [COD: 00-901-4359], and calcium carbonate (CaCO3) [COD: 00-101-0928].

Other crystalline phase observed in both the powder and pellet was tri dialuminium silicate (Ca3Al2(SiO4)3) [COD: 00-101-1055].

### Analysis of biocompatibility

The immediate cytotoxicity in the experimental cement (Group 1) showed a significant decrease at 48 h and a significant increase at 72 h when compared to MTA (*p* < 0.05) (Table 2). However, no significant difference was observed at 24 h between the 2 two groups (*p* < 0.001).

**Table 2:** Relative immediate cell viability.

	Mean ± SD		*P* value	95% Confidence Interval of the difference	
	Group 1	Group 2		Lower	Upper
24 h	0.91 ± 0.02	0.91 ± 0.08	0.752	−0.01	0.01
48 h	0.87 ± 0.01	0.85 ± 0.00	0.001	0.00	0.02
72 h	0.61 ± 0.01	0.62 ± 0.00	0.011	−0.01	0.00

The cytotoxicity of the set samples in the experimental group (Group 1) showed a significant decrease at 24 h followed by a significant increase at 48 h (*p* < 0.001). There was no significant difference in the cytotoxicity of the set samples between the groups at 72 h (*p* > 0.001) (Table 3).

**Table 3:** Relative cell viability in set samples.

	Mean ± SD		*P* value	95% Confidence Interval of the difference	
	Group 1	Group 2		Lower	Upper
24 h	1.13 ± 0.02	0.99 ± 0.01	0.001	0.13	0.16
48 h	0.77 ± 0.10	1.00 ± 0.05	0.001	−0.28	−0.16
72 h	0.94 ± 0.04	0.93 ± 0.04	0.390	−0.01	0.04

### Analysis of antibacterial activity

A significant increase in the antibacterial activity was observed in Group 1 when compared to Group 2 (*p* < 0.001) (Table 4).

**Table 4:** Antibacterial activity of the cement samples (in mm).

Mean ± SD		*P* value	95% Confidence Interval of the difference	
Group 1	Group 2		Lower	Upper
3.50 ± 0.51	1.31 ± 0.47	<0.001	1.82	2.54

## Discussion

According to the previous research reports, the constituent phase of MTA is predominantly tricalcium silicate, followed by dicalcium silicate and tricalcium aluminate. The experimental calcium silicate-based cement (nano CS) fabricated using a solid-state reaction at a temperature of 100 °C showed the presence of dicalcium silicate and tricalcium aluminate in the unhydrated sample (Fig. 2). Tricalcium silicate was not formed in the hydration reaction as the production of this material was performed at a low temperature. Moreover, tricalcium silicate primarily contributes to the early strength of the material. For root-end filling materials, compressive strength is not a major concern as minimal forces are applied in the root-end region (Kang et al., 2021). On the other hand, dicalcium silicate cement as a root-end filling material has been shown to manifest enormous apatite-forming activity and lesser degradation in acidic environments (Chiang & Ding, 2013). Concerning biocompatibility, dicalcium silicate cement has been proven to be significantly superior to traditional MTA (Chiang & Ding, 2010). The biological evaluation of dicalcium silicate cement showed adequate properties in terms of bioactivity and biocompatibility for its application as a root-end filling and pulp capping material (Gou et al., 2005; Liangjiao et al., 2015; Wu et al., 2015; Chen et al., 2009; Chen, Shie & Ding, 2011; Gandolfi et al., 2010).

The hydration rate of tricalcium aluminate is known to be more rapid among the principal components of Portland cement (Saghiri et al., 2017). The literature on cement chemistry shows that it speeds up the hydration process and enhances the short-term compressive strength of tricalcium silicate/tricalcium aluminates composites in comparison to pure tricalcium silicate (Taylor, 1997; Ramachandran, 1996).

The addition of a radio-opacifier is a requirement for all hydraulic endodontic cements to comply with the existing standard (Camilleri, 2020). This criterion had been fulfilled with the addition of bismuth oxide and the crystalline phases of bismite were observed in both hydrated and unhydrated samples. In addition, a peak of calcium hydroxide was also observed in the hydrated sample which signified a greater rate of reaction (Camilleri, Formosa & Damidot, 2013).

The experimental cement combined with the silver nanoparticles needs to be in close proximity to the oral and periapical tissues (Dsouza et al., 2020b). Owing to the utmost importance of the biological effects of the retrograde material, investigation of the biological reaction of this material with the periapical tissues is quintessential. In the current study, the MTT test was performed in living, metabolically active cells to assess biocompatibility (Bossù et al., 2021). The amount of formazan formed during the reaction determines the viability of the cells (Augustine et al., 2018). The experimental cement showed favorable biocompatibility when compared to MTA (Tables 2 and 3). Also, the biocompatibility at 72 h was similar to that of MTA. Our results are consistent with the previous studies of Zand et al. (2016) wherein MTA incorporated with 1 wt% silver nanoparticles did not affect the inflammatory reaction of subcutaneous tissue in rat models. Also, Dsouza et al. (2020b) proved that silver or gold nanoparticles added to calcium silicate-based cements did not influence the overall biocompatibility of those materials when human lymphocytes were used.

Another attribute of the biological property of root-end filling materials to be considered is the antibacterial activity. The eradication of micro-organisms is key to overall treatment success; hence, some antimicrobial activity is ideally required in root-end filling materials (Corrêa et al., 2015). The popular calcium silicate-based root-end filling material MTA has shown inconsistent results concerning antibacterial activity, owing to its alkaline pH. Therefore, an add-on ingredient that strongly amplifies the inhibitory effects of MTA, while preserving its biocompatibility, would be desirable (Bahador et al., 2014). Silver nanoparticles (1 wt%) were incorporated as the additive to impart antibacterial activity. They have been shown to exhibit an antimicrobial effect even at a low level, due to their small size but large surface area (Priyadarsini, Mukherjee & Mishra, 2018). There was a significant increase in antibacterial activity in the experimental group when compared to ProRoot MTA (Table 4). Our results are in agreement with previous literature (Bahador et al., 2014; Jonaidi-Jafari, Izadi & Javidi, 2016; Samiei et al., 2013), who concluded that nanosilver could serve as an excellent additive to MTA and other calcium silicate-based cements to protect against anaerobic endodontic-periodontal micro-organisms. Moreover, Vazquez-Garcia et al. (2016) observed that adding silver nanoparticles to calcium silicate cements favored the physicochemical and mechanical properties of the materials.

## Conclusions

The experimental silver nanoparticle incorporated cement, manufactured using the solid-state reaction technique, yielded a fine powder with the investigation of the hydration characteristics confirming the presence of dicalcium silicate, suggesting that the material was calcium silicate-based. The further findings of this research demonstrated that the experimental calcium silicate-based cement almost satisfies the ideal requirements of the root-end filling material with regard to biological properties. Thus, this material could be considered a promising alternative to MTA, with favorable biocompatibility and increased antibacterial activity.

##  Supplemental Information

10.7717/peerj.14632/supp-1 Supplemental Information 1 XRD data of the experimental powder sample

10.7717/peerj.14632/supp-2 Supplemental Information 2 XRD data of the experimental powder and liquid mixed together (pellet)

10.7717/peerj.14632/supp-3 Supplemental Information 3 Antibacterial activity (zone of inhibition in mm)

10.7717/peerj.14632/supp-4 Supplemental Information 4 Cell viability data

## Additional Information and Declarations

### Data Availability

The following information was supplied regarding data availability: The raw measurements are available in the Supplementary Files.
